# Long-Term Secondary Prevention Gaps Among Young Survivors of Acute Myocardial Infarction in China: A Hospital-Based Cross-Sectional Study

**DOI:** 10.3390/healthcare14203407

**Published:** 2026-10-13

**Authors:** Yu-Guo Xue, Jin-Wen Wang, Xue-Yao Yang, Min Zhang, Hui-Juan Zuo

**Affiliations:** 1 Department of Cardiology, Beijing Anzhen Hospital, Capital Medical University, Beijing 100029, China; xueyuguo2006@163.com (Y.-G.X.); yxy1231994@163.com (X.-Y.Y.); 2 Department of Community Health Research, Beijing Institute of Heart Lung and Blood Vessel Diseases, Beijing Anzhen Hospital, Capital Medical University, Beijing 100029, China; wang-jin-wen@163.com

**Keywords:** premature acute myocardial infarction, secondary prevention, lifestyle modification, real-world study

## Abstract

**Background**: Premature acute myocardial infarction (AMI) has become an important public health challenge. Young survivors face prolonged residual cardiovascular risk, yet real-world implementation of long-term secondary prevention across medication use, health behaviors, and risk-factor monitoring remains insufficiently characterized in China. **Methods**: In this hospital-based cross-sectional study, we included 1377 young AMI survivors aged 18–44 years who attended follow-up at Beijing Anzhen Hospital between 2023 and 2025. Current use of major secondary prevention medications, achievement of four health-behavior targets (smoking abstinence/cessation, physical activity, diet, and sleep), and lipid monitoring during the preceding 12 months were assessed using standardized interviews and medical records. Recurrent myocardial infarction (MI) and repeat coronary revascularization occurring between discharge after the index AMI and the study visit were retrospectively identified. Multivariable logistic regression was used to examine associations between secondary-prevention implementation domains and recurrent events. **Results**: Participants had a mean age of 39.1 years, and 95.9% were male. Recurrent MI occurred in 9.2% and repeat revascularization in 13.7%. The proportion concurrently using four major medication classes decreased from 50.0% at discharge to 25.9% at follow-up. Overall, 45.1% achieved good behavioral implementation, defined as meeting at least three of four ideal behaviors. In fully adjusted models, good behavioral implementation was associated with lower odds of recurrent MI (odds ratio [OR] 0.49, 95% confidence interval [CI] 0.32–0.73) and repeat revascularization (OR 0.72, 95% CI 0.52–1.00). Concurrent use of all four medication classes and frequent lipid monitoring were not independently associated with recurrent outcomes. **Conclusions**: Among young AMI survivors in China, substantial gaps in long-term secondary prevention were observed, particularly for healthy behaviors. These findings support the public health importance of strengthening lifestyle-oriented and multidisciplinary follow-up strategies for young survivors of AMI. Because exposure and outcome periods overlapped partially, findings should be interpreted as associational rather than causal.

## 1. Introduction

Cardiovascular disease (CVD) remains a leading cause of morbidity and mortality worldwide. Although age-standardized mortality from ischemic heart disease has declined in many settings, acute myocardial infarction (AMI) is increasingly affecting younger adults. This trend has important public health implications, as young survivors may live for decades with elevated residual cardiovascular risk and ongoing needs for secondary prevention [1,2]. Compared with older patients, younger individuals with AMI often have distinct risk profiles characterized by smoking, obesity, and metabolic abnormalities, highlighting the need for sustained long-term management after the acute event [3,4,5].

Secondary prevention after AMI requires more than acute treatment alone and depends on the long-term implementation of evidence-based medications, healthy behaviors, and risk-factor monitoring. Guideline-recommended medications, including antiplatelet agents, statins, beta-blockers, and renin–angiotensin system inhibitors when clinically indicated, reduce the risk of recurrent cardiovascular events [6,7]. Health behaviors such as smoking abstinence, regular physical activity, healthy dietary patterns, and adequate sleep are also central to cardiovascular health and are emphasized in contemporary prevention frameworks, including the American Heart Association’s Life’s Essential 8 [8,9,10,11].

Despite these advances, important gaps remain in the real-world implementation of secondary prevention after AMI [7]. Previous studies have shown that the use of preventive medications and adherence to healthy lifestyle recommendations often decline over time following hospital discharge [12,13,14,15]. Younger survivors may face additional barriers to sustained secondary prevention, including competing work and family responsibilities, psychosocial stress, and reduced perceived vulnerability after symptom relief or coronary intervention. As a result, suboptimal implementation may persist across multiple domains of secondary prevention, including medication use, health behaviors, and follow-up monitoring.

Evidence on multidomain secondary prevention among young AMI survivors remains limited, particularly in China, where the burden of premature coronary artery disease is substantial. In particular, it is unclear how medication use, health behaviors, and lipid monitoring are implemented simultaneously in routine care among young survivors of AMI, which patient characteristics are associated with better implementation across these domains, and whether these patterns are associated with recurrent cardiovascular events.

Therefore, this study aimed to characterize the real-world implementation of multidomain secondary prevention among young survivors of AMI in China, including medication use, health behaviors, and lipid monitoring; to examine clinical characteristics associated with better implementation across these domains; and to explore the associations between these implementation patterns and recurrent cardiovascular events in routine clinical practice.

## 2. Methods

### 2.1. Study Design and Population

This study was a single-center, real-world, cross-sectional observational study conducted at Beijing Anzhen Hospital, Capital Medical University. All study data were obtained during a single outpatient follow-up visit.

Adherence patterns were assessed based on participants’ typical behaviors and medication use during the 12 months preceding the study visit. Clinical outcomes, including recurrent myocardial infarction (MI) and repeat coronary revascularization, were retrospectively ascertained from the time of index AMI discharge to the study visit using the hospital’s electronic medical record system.

Because adherence information and clinical outcomes were collected within partially overlapping observation periods, the temporal sequence between adherence behaviors and clinical events cannot be strictly established. Therefore, this study was designed primarily to describe real-world patterns of secondary prevention and to explore associations between adherence patterns and clinical outcomes rather than to determine causal relationships.

We consecutively enrolled young adults with a confirmed diagnosis of acute myocardial infarction (AMI) who attended the cardiology outpatient clinic between January 2023 and May 2025. Eligible participants met the following inclusion criteria: (1) age between 18 and 44 years at the time of the index AMI diagnosis; and (2) the index AMI event occurring at least 12 months before study enrollment. Patients were excluded if they had incomplete information on key health behaviors or medication use, or if they had severe non-cardiac comorbidities that substantially limited life expectancy.

The study was approved by the Medical Ethics Committee of Beijing Anzhen Hospital, Capital Medical University (Approval No. KS2022071; approved on 28 September 2022). Written informed consent was obtained from all participants prior to enrollment.

### 2.2. Data Collection

Data were collected through standardized face-to-face interviews using a structured questionnaire (see Supplementary File S2), physical measurements, and extraction of electronic medical records (EMR) by trained investigators following a predefined protocol. Information collected included demographic characteristics, clinical comorbidities, medication use, health behaviors, and monitoring practices.

For each participant, recurrent MI and repeat revascularization events occurring between the index discharge and the study visit were identified from the hospital’s EMR system. Information on adherence—including medication use, health behaviors, and monitoring frequency—was assessed based on the participant’s typical pattern during the 12 months preceding the study visit. Although some individual items on the structured questionnaire did not explicitly state this timeframe, all trained investigators were strictly instructed to anchor the participants’ recall to their typical, sustained lifestyle patterns and medication usage over the preceding 12 months, avoiding descriptions of transient habits on the day of the interview.

#### Clinical Comorbidities

Clinical comorbidities were defined as follows:

- Hypertension: current use of antihypertensive medication or clinical blood pressure ≥ 140/90 mmHg [16].
- Diabetes Mellitus: previous physician diagnosis of diabetes or current use of glucose-lowering medication [17].
- Obesity: body mass index (BMI) >24 kg/m^2^ according to recommended cut-offs for Asian populations.
- Suboptimal LDL-C level: defined as LDL-C ≥1.8 mmol/L at the study visit [6].

### 2.3. Definitions of Medication Use, Behavioral Implementation, and Monitoring

#### 2.3.1. Definition of Medication Use

Medication use was assessed based on four major secondary prevention drug classes commonly recommended after AMI: antiplatelet agents (aspirin or P2Y12 inhibitors), statins, beta-blockers, and renin–angiotensin system inhibitors (angiotensin-converting enzyme inhibitors or angiotensin receptor blockers [ACEI/ARB]).

To clearly distinguish between two distinct time points, we recorded both the medical prescriptions issued at the index AMI discharge and participants’ self-reported current medication use at the study visit to capture longitudinal changes in treatment. Because all participants were enrolled at least 12 months after the index AMI, treatment modifications during this interval were anticipated and could reflect clinically appropriate changes in therapy. In particular, the duration of dual antiplatelet therapy (DAPT) is individualized, and the transition from DAPT to single antiplatelet therapy (SAPT) is routinely recommended after approximately 12 months in appropriate patients. Accordingly, the use of at least one antiplatelet agent at the study visit was classified as positive current antiplatelet use.

The pharmacological composite indicator was derived exclusively from the self-reported current medication status at the study visit. We defined “concurrent four-class medication use” as the active use of all four drug categories listed above. Importantly, detailed information regarding longitudinal persistence, pharmacy refill history, treatment interruptions, dose reductions, or long-term compliance was not systematically available. Therefore, our assessment fundamentally reflects current medication exposure or treatment implementation at the time of the visit, rather than true longitudinal medication adherence. Furthermore, detailed clinical data required to adjudicate individualized treatment appropriateness—such as specific contraindications, drug intolerance, or physician-directed discontinuation decisions (e.g., stopping beta-blockers following the recovery of left ventricular function)—were not collected. Consequently, the absence of any individual drug class reflects reported non-use and was explicitly not adjudicated as patient non-adherence, as the indication for individual medications varies according to changing clinical characteristics and tolerance [18].

#### 2.3.2. Behavior Implementation

Health behaviors were evaluated using a study-specific structured questionnaire developed based on the American Heart Association (AHA) Life’s Essential 8 (LE8) framework and adapted to Chinese lifestyle guidelines [11,19,20]. Four key behavioral domains were assessed, with standardized scoring and classification rules predefined as follows:

Nicotine Exposure: Never smoked or successfully quit smoking for more than 12 months.

Physical Activity: Physical activity duration was recorded through structured interviewer-administered questions and categorized by intensity (moderate vs. vigorous based on MET values). Participants meeting weekly thresholds (≥150 min/week moderate-intensity aerobic activity or ≥75 min/week vigorous-intensity activity) were defined as having adequate physical activity.

Diet Quality: Dietary adherence was scored from 0 to 7 based on a simplified instrument adapted from the AHA Life’s Essential 8 (LE8) framework and Chinese cardiovascular secondary prevention guidelines [11,20,21]. Participants meeting ≥4 of the seven items were classified as having a healthy diet. The seven criteria were defined as follows:

(1) Consumption of whole grains and mixed legumes ≥3 times per week;
(2) Vegetable intake ≥300 g per day;
(3) Fruit intake ≥200 g per day;
(4) Red meat consumption ≤75 g per day;
(5) Reduced intake of fats, operationalized as self-reported sustained reductions over the preceding 12 months in dietary sources of unhealthy fats (specifically assessed via three sub-items: reduced consumption of animal-derived foods, deep-fried foods, and high-oil pastries);
(6) Reduced intake of salt, operationalized as self-reported sustained reductions in sodium intake over the preceding 12 months (specifically assessed via three sub-items: self-perceived preference for less salty food, reduced use of salt during home cooking, and decreased consumption of pickled or high-sodium foods);
(7) No or moderate alcohol consumption (≤25 g/day for men, ≤15 g/day for women).

The threshold of ≥4 criteria was pre-specified to ensure a clinically meaningful and statistically viable group distribution. Applying the stringent original AHA LE8 dietary standard (which requires meeting ≥15 of 16 items) yields an extremely low prevalence of ideal diet (historically <1% to 7% in general populations), which would render the behavioral subgroup statistically underpowered in this young post-AMI cohort. The ≥4/7 cutoff provides a pragmatic differentiation of dietary management aligned with real-world secondary prevention capabilities.

Sleep Health: Average nightly sleep duration of 7–9 h.

A composite behavioral score was constructed by summing the number of achieved criteria across the four domains (range: 0–4). The threshold for “good behavioral adherence” was defined as meeting ≥3 of the 4 domains. This was a prespecified, study-specific pragmatic threshold conceptually informed by the American Heart Association’s Life’s Essential 8 (LE8) framework. Although the validated LE8 scoring system applies a ≥80% threshold to define high cardiovascular health, our dichotomized classification was established specifically for the present analysis. It was chosen to identify participants implementing the majority of favorable health behaviors while maintaining sufficient statistical power for multivariable comparative analyses.

#### 2.3.3. Monitoring Patterns

Participants were queried about the frequency of blood pressure, blood glucose, and lipid testing, including both home self-monitoring and hospital-based laboratory testing. Frequent lipid monitoring was defined as undergoing venous LDL-C testing at least once every three months during follow-up. This threshold was pre-specified based on dyslipidemia management guidelines, which recommend lipid surveillance every 1 to 3 months for high-risk post-MI patients to evaluate LDL-C target attainment and guide medication titration [21].

### 2.4. Outcome Definitions

The primary outcomes were recurrent myocardial infarction (re-MI) and unplanned, ischemia-driven repeat coronary revascularization, including percutaneous coronary intervention (PCI) or coronary artery bypass grafting (CABG). Only events occurring after discharge from the index MI hospitalization and before the study visit were considered. Events occurring during the index hospitalization were not counted as recurrent MI.

All planned or staged PCI procedures pre-scheduled during the index AMI hospitalization were strictly excluded from endpoint counting.

To ensure accurate outcome ascertainment and minimize recall bias, events were identified via a dual verification mechanism combining extraction from the hospital’s central electronic medical record (EMR) system with structured face-to-face patient interviews. Clinical events treated within our center were verified using internal hospital discharge summaries, procedural records, and angiographic reports. For clinical events occurring at external healthcare institutions, relevant diagnoses and procedural details were further confirmed using outside hospital discharge summaries and interventional records provided by participants.

### 2.5. Statistical Analysis

Continuous variables are presented as mean ± standard deviation (SD), or median with interquartile range (IQR), as appropriate, and categorical variables are summarized as frequencies and percentages. Differences between independent groups were assessed using Student’s *t*-test or the Mann–Whitney U test for continuous variables and the chi-square test for categorical variables. To compare paired medication use data collected at two time points (index AMI hospitalization and outpatient follow-up), McNemar’s test was applied for binary individual drug class variables, and the Wilcoxon signed-rank test was used for the ordinal count of concurrent drug classes (range 0–4).

Associations between secondary preventive measures and clinical outcomes were evaluated via multivariable logistic regression models. This approach was selected due to the cross-sectional study design and the lack of uniform time-to-event data for all participants. Three hierarchical regression models were constructed: Model 1 (unadjusted); Model 2 adjusted for demographic and baseline clinical covariates (age, sex, AMI subtype, multivessel disease, time from index MI to enrollment); and Model 3 with further adjustment for traditional cardiovascular risk factors (hypertension, diabetes mellitus, BMI, LDL-C) plus mutual adjustment across all secondary prevention adherence domains. For multivariable adjustment, BMI was analyzed as a binary categorical variable indicating overweight/obesity status (BMI < 24 kg/m^2^ versus BMI ≥ 24 kg/m^2^). All covariates were pre-specified based on clinical relevance and the established cardiovascular literature, adhering to the event-per-variable rule to prevent model overfitting. Regression results are reported as odds ratios (ORs) with 95% confidence intervals (CIs). All analyses relied on complete case data; participants with missing key exposure or outcome values were excluded from corresponding regression models, and multiple imputation was not performed.

Missing data were systematically assessed and found to be limited exclusively to the BMI covariate, with valid measurements available for 1260 (91.5%) participants. All primary exposures, outcomes, and other clinical covariates were 100% complete. Therefore, multiple imputation was not performed, and analyses relied on complete-case data. Consequently, Models 1 and 2 included all 1377 participants, whereas Model 3 included 1260 participants. The exact sample sizes retained in each multivariable logistic regression model are explicitly stated in the footnotes of the corresponding result tables to enhance analytical transparency.

A sensitivity analysis was conducted to mitigate reverse causation bias by excluding participants who experienced recurrent cardiovascular events within the 12-month behavioral recall window before the study visit. Additionally, a second sensitivity analysis restricting the cohort strictly to male participants was performed to evaluate the stability of our findings in this highly male-dominated population. Prior to finalizing the multivariable models, multicollinearity was assessed using the Variance Inflation Factor (VIF), with a VIF < 5 indicating the absence of significant collinearity. Model calibration and goodness-of-fit were evaluated using the Hosmer–Lemeshow test.

All statistical analyses were performed with SPSS version 27.0 (IBM Corp., Chicago, IL, USA) and R version 4.2.3 (R Foundation for Statistical Computing, Vienna, Austria). A two-sided *p*-value < 0.05 was defined as the threshold for statistical significance.

## 3. Results

### 3.1. General Characteristics

A total of 1377 young patients with AMI were included in this study. The cohort was predominantly male (95.9%; *n* = 1321), with a mean age at first diagnosis of 39.1 ± 4.4 years. ST-segment elevation myocardial infarction (STEMI) accounted for 77.6% (*n* = 1069) of initial presentations, and multi-vessel disease was present in 42.9% (*n* = 591) of patients. During the observation period, recurrent MI occurred in 9.2% (*n* = 126) of patients, while 13.7% (*n* = 188) underwent repeat revascularization. The median time from index AMI to study visit was 6 years (IQR: 4–8 years).

Univariate analysis identified several factors significantly associated with clinical outcomes (all *p* < 0.05). For recurrent MI, these factors included the type of MI, multi-vessel disease, hypertension, diabetes, non-smoking/cessation status, adherence to a healthy diet, and the use of statins and beta-blockers. Notably, the proportion of multivessel disease was significantly higher in patients with recurrent MI than in those without (51.6% vs. 42.0%, *p* = 0.039). For repeat revascularization, significant correlates included multi-vessel disease, hypertension, diabetes, healthy diet adherence, and the use of antiplatelet drugs, statins, and beta-blockers. Detailed results are presented in Table 1.

### 3.2. Patterns of Secondary Preventive Measures for Coronary Artery Disease

#### 3.2.1. Utilization of Guideline-Recommended Secondary Prevention Medications

Current use of guideline-recommended secondary preventive medications declined substantially from index AMI discharge to the study visit (Table 2). At the time of index AMI hospitalization discharge, prescription rates were 92.1% for dual antiplatelet therapy, 87.1% for statins, 78.2% for beta-blockers, and 59.1% for ACEI/ARBs; half (50.0%) of participants were prescribed all four drug classes simultaneously. At the later study visit, the corresponding utilization rates fell to 86.6%, 79.7%, 59.8%, and 35.7%, while the proportion of patients using the complete four-drug regimen decreased to 25.9%. Paired comparisons via McNemar’s and Wilcoxon signed-rank tests revealed statistically significant reductions in both individual drug class use and quadruple combined medication use from discharge to the study visit (all *p* < 0.001). Notably, these declines do not necessarily represent poor medication use, as long-term treatment de-escalation (e.g., switching from dual to single antiplatelet therapy) and dose or class discontinuation may reflect individualized guideline-aligned care.

The antiplatelet regimen was adjusted dynamically: dual antiplatelet therapy was administered upon initial AMI diagnosis, and switched to monotherapy (aspirin, clopidogrel, or ticagrelor) during the outpatient follow-up phase.

#### 3.2.2. Behavioral Implementation of Guideline-Recommended Health Behaviors

At the follow-up, rates of meeting ideal health-behavior targets were as follows: 54.9% for non-smoking/cessation, 74.8% for regular moderate physical activity, 58.3% for a healthy diet, and 43.5% for adequate sleep. Stratified by the cumulative number of achieved behavioral targets, 23.0% (*n* = 317) of patients met 0–1 targets, 31.9% (*n* = 439) met 2 targets, 31.3% (*n* = 431) met 3 targets, and 13.8% (*n* = 190) met all 4 targets. Overall, 45.1% (*n* = 621) of patients achieved “Good behavioral implementation” (meeting ≥3 ideal health-behavior targets).

#### 3.2.3. Risk-Factor Monitoring Patterns

Regarding lipid profile monitoring, the most common testing intervals were 1–3 months (45.8%) and 3–6 months (45.6%), with no significant correlation between testing frequency and the achievement of target LDL-C levels (*p* = 0.055). For blood pressure monitoring, 64.6% of patients measured their blood pressure at least once a month, and hypertensive patients had significantly more frequent monitoring than non-hypertensive patients (*p* < 0.001). Blood glucose monitoring frequency was evenly distributed: 30.9% monthly, 33.2% every 1–3 months, and 35.9% every 3–6 months, with diabetic patients monitoring significantly more frequently than non-diabetic patients (*p* < 0.001). Detailed data are presented in Table 3.

### 3.3. Characteristics Associated with Secondary-Prevention Implementation

Patient characteristics showed distinct correlates of adherence across different dimensions of secondary prevention (Table 4). Higher medication use was more common among men and among patients with hypertension or a history of recurrent MI (*p* < 0.05). Better adherence to behavioral modifications was more frequently observed in women, older patients, and those with an initial diagnosis of NSTEMI (all *p* < 0.05). Conversely, participants with prior recurrent MI or repeat revascularization exhibited lower rates of good behavioral implementation (*p* < 0.05). Older age and a history of diabetes were also associated with higher adherence to lipid testing (*p* < 0.05).

### 3.4. Interrelationships Among Medication, Behavioral, and Frequent Lipid Monitoring

**Figure 1:**
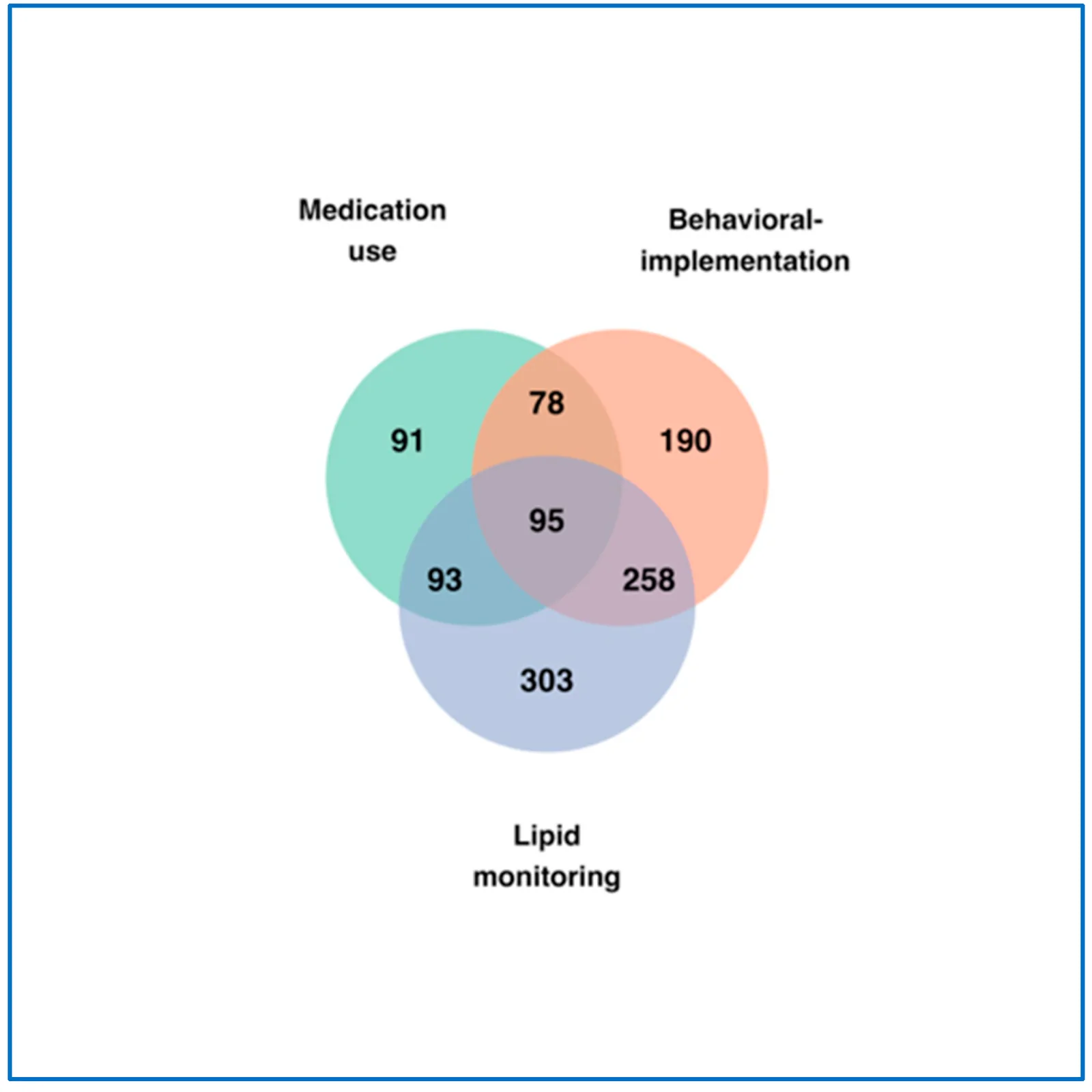
Overlap of three secondary prevention implementation domains among young AMI survivors. Notes: Venn diagram illustrating participant overlap across three secondary-prevention implementation domains (*n* = 1377). Domains include medication use, behavioral implementation, and lipid monitoring. Values within each segment indicate participant counts for exclusive or overlapping subgroups. In total, 269 participants did not satisfy the criteria for any of the three secondary-prevention domains and are not visualized in this Venn diagram.

No significant associations were observed among the three adherence domains. The proportion of participants with good behavioral implementation was comparable between those with and without full medication use (48.5% vs. 43.9%, *p* = 0.138). When stratified by behavioral implementation status, frequent lipid monitoring was 52.4% among participants with poor behavioral implementation and 56.8% among those with good behavioral implementation (*p* = 0.098). Frequent lipid monitoring was 55.0% in participants without full medication use and 52.7% in those with full medication use, with no statistically significant difference (*p* = 0.445). Detailed results are shown in Figure 1 and Supplementary Table S1.

### 3.5. Associations Between Adherence Domains and Clinical Outcomes

Model diagnostics for the fully adjusted models confirmed excellent statistical performance. No significant multicollinearity was detected among the covariates (all VIFs ≤ 1.113). Furthermore, the Hosmer–Lemeshow test indicated good model calibration and adequate goodness-of-fit for both the recurrent MI model (*p* = 0.471) and the repeat revascularization model (*p* = 0.537).

#### 3.5.1. Medication Use

In unadjusted logistic regression, adherence to all four guideline-directed medication classes was associated with higher odds of recurrent MI (OR 1.546, 95% CI 1.047–2.283). This association remained similar after adjustment for demographics and clinical presentation. In the fully adjusted model, medication use was not significantly associated with the odds of recurrent MI (OR 1.472, 95% CI 0.979–2.212; *p* = 0.063) or repeat revascularization (OR 1.222, 95% CI 0.861–1.736; *p* = 0.262). As observed in baseline characteristic comparisons, patients with recurrent events exhibited a higher prevalence of multivessel disease and hypertension.

To address the varying clinical indications and effects of different pharmacological agents, we conducted an additional analysis evaluating each of the four medication classes separately within the fully adjusted model (Supplementary Table S2). For recurrent MI, none of the individual medication classes—antiplatelet drugs (adjusted OR = 1.068, 95% CI: 0.480–2.379), statins (adjusted OR = 1.428, 95% CI: 0.724–2.817), beta-blockers (adjusted OR = 1.434, 95% CI: 0.923–2.227), or ACEI/ARBs (adjusted OR = 1.064, 95% CI: 0.706–1.604)—demonstrated a statistically significant association. However, for repeat revascularization, the current use of beta-blockers was significantly associated with higher odds of the event (adjusted OR = 1.652, 95% CI: 1.106–2.467, *p* = 0.014), whereas the associations for antiplatelet drugs, statins, and ACEI/ARBs remained non-significant.

#### 3.5.2. Behavioral Implementation

Good behavioral implementation, pre-defined as meeting the criteria for at least three of the four behavioral domains, was associated with lower odds of both clinical endpoints (Table 5). In the fully adjusted model (Model 3), good behavioral implementation was associated with significantly lower odds of recurrent MI (adjusted OR = 0.487, 95% CI: 0.324–0.731; *p* < 0.001). For repeat revascularization, a similar inverse association was observed (adjusted OR = 0.723, 95% CI: 0.522–1.001; *p* = 0.050).

To further examine the robustness and specific components driving this composite behavioral measure, two additional sets of analyses were performed (Supplementary Table S3).

First, the number of implemented behavioral domains was analyzed as an ordinal variable (0–1, 2, 3, and 4 domains), using 0–1 domains as the reference category. A clear graded inverse association was observed for both outcomes. Compared with participants implementing 0–1 behavioral domains, those achieving three domains had significantly lower odds of recurrent MI (adjusted OR = 0.515, 95% CI: 0.313–0.849; *p* = 0.009), while those achieving all four domains demonstrated substantially lower odds (adjusted OR = 0.166, 95% CI: 0.063–0.436; *p* < 0.001). A corresponding graded pattern was observed for repeat revascularization, with significantly lower odds among participants implementing three (adjusted OR = 0.665, 95% CI: 0.436–0.953; *p* = 0.028) and four behavioral domains (adjusted OR = 0.438, 95% CI: 0.236–0.811; *p* = 0.009).

Second, to disentangle the independent associations of each specific lifestyle factor, all four individual behavioral domains were entered simultaneously into the fully adjusted multivariable logistic regression model. Smoking cessation was independently associated with lower odds of recurrent MI (adjusted OR = 0.561, 95% CI: 0.382–0.825; *p* = 0.003) and repeat revascularization (adjusted OR = 0.723, 95% CI: 0.526–0.995; *p* = 0.046). Similarly, a healthy diet was independently associated with lower odds of recurrent MI (adjusted OR = 0.622, 95% CI: 0.423–0.913; *p* = 0.016) and repeat revascularization (adjusted OR = 0.710, 95% CI: 0.514–0.982; *p* = 0.038). Physical activity showed a non-significant inverse trend with recurrent MI (adjusted OR = 0.686, 95% CI: 0.451–1.044; *p* = 0.078). Adequate sleep was not materially associated with either endpoint.

#### 3.5.3. Frequent Lipid Monitoring

Frequent lipid monitoring (defined as LDL-C testing at least once every 3 months) was not significantly associated with the odds of recurrent MI (adjusted OR = 0.928, 95% CI: 0.632–1.362; *p* = 0.702) or repeat revascularization (adjusted OR = 1.059, 95% CI: 0.767–1.461; *p* = 0.729) in the fully adjusted models (Table 5).

Event-date information was complete for all recorded recurrent clinical events. Only 5 patients experienced a recurrent event (recurrent MI or repeat revascularization) within the 12-month recall window prior to the study visit. In pre-specified sensitivity analyses excluding these 5 participants (*n* = 1372), the inverse association between good behavioral implementation and lower odds of recurrent MI remained virtually unchanged and statistically significant (adjusted OR = 0.517, 95% CI: 0.343–0.799, *p* = 0.002). For repeat revascularization, the point estimate was materially similar to the primary analysis but no longer reached conventional statistical significance (adjusted OR = 0.747, 95% CI: 0.537–1.038, *p* = 0.083). Full results of this sensitivity analysis are presented in Supplementary Table S4.

Finally, a sensitivity analysis restricting the cohort strictly to male participants (*n* = 1321) yielded results that were virtually identical to the primary analyses, with good behavioral implementation remaining strongly associated with lower odds of recurrent MI and repeat revascularization (detailed in Supplementary Table S5).

### 3.6. Associations Between Composite Adherence and Clinical Outcomes

We further examined overall adherence by integrating medication, behavioral, and monitoring domains. Patients were categorized into four groups based on the number of adherence domains achieved (0–3), using the 0-domain group as reference.

No clear association pattern or dose–response relationship was observed across models (Supplementary Table S1).

## 4. Discussion

This hospital-based cross-sectional study provides a real-world assessment of multidomain secondary prevention among young survivors of acute myocardial infarction in China. We observed substantial gaps in the long-term implementation of secondary prevention, particularly in healthy behaviors, despite relatively common use of major secondary prevention medications in this tertiary-care cohort. Better implementation of healthy behaviors—including smoking abstinence and cessation, regular physical activity, healthy diet, and adequate sleep—was associated with lower odds of recurrent myocardial infarction, whereas current use of all four major medication classes and frequent lipid monitoring were not independently associated with recurrent outcomes in adjusted analyses. We also found limited overlap across medication use, health behaviors, and monitoring practices, suggesting that secondary prevention in young AMI survivors is multidimensional and that better implementation in one domain does not necessarily indicate better implementation in others.

Taken together, these findings highlight the multidimensional nature of secondary prevention in young patients with coronary disease. While pharmacological therapy remains a cornerstone of post-AMI management, our results suggest that sustained lifestyle engagement represents an important complementary component of long-term cardiovascular risk-management strategies. From a public health perspective, these findings underscore the need to strengthen behavioral and lifestyle interventions as part of comprehensive secondary prevention strategies for young AMI survivors [22]. Because adherence patterns and clinical outcomes were assessed within partially overlapping observation windows, these findings should be interpreted as exploratory associations rather than causal effects.

### 4.1. Healthy Behaviors and Long-Term Secondary Prevention in Young AMI Survivors

Healthy lifestyle behaviors have consistently been shown to play an essential role in cardiovascular prevention. Our findings are broadly consistent with this body of evidence. In this cohort of young AMI survivors, adherence to multiple healthy behaviors was associated with significantly lower odds of recurrent myocardial infarction in adjusted analyses. Although causal relationships cannot be established in the present study, these findings suggest that lifestyle behaviors represent an indispensable component of long-term cardiovascular risk management.

Young patients with premature coronary artery disease may particularly benefit from sustained lifestyle engagement because behavioral risk factors—such as smoking, poor diet, and physical inactivity—often play a prominent role in the development of early atherosclerosis [23]. Moreover, unlike older patients, young survivors face decades of future cardiovascular risk. Therefore, maintaining healthy behaviors after an index cardiovascular event has profound implications for long-term risk management.

Notably, our component analyses revealed a clear inverse association between the number of adopted healthy behaviors and recurrent events, with smoking cessation and a healthy diet emerging as the primary independent drivers. Despite these potential benefits, adherence to recommended lifestyle behaviors remains suboptimal. In our study, fewer than half of participants achieved good behavioral implementation. Together, these findings highlight persistent gaps in lifestyle counseling and behavioral implementation within real-world secondary prevention programs [12,13].

### 4.2. Interpretation of Pharmacological Treatment Implementation and Medication Use Patterns

The absence of a significant inverse association between concurrent four-class medication use and recurrent events warrants careful interpretation. In this observational cohort, medication use primarily reflects current treatment implementation rather than longitudinal adherence. This counterintuitive neutral association may be explained, at least in part, by confounding by indication, unmeasured residual confounding, and evolving individualized pharmacotherapy. Modern cardiovascular care has shifted from a rigid “one-size-fits-all” approach toward personalized regimens, where discontinuation of beta-blockers in selected patients with recovered left ventricular ejection fraction (LVEF) or transition to single antiplatelet therapy may represent appropriate physician-directed de-escalation.

Our component analysis of individual medication classes further supports the possibility of confounding by indication: current use of beta-blockers was independently associated with higher odds of repeat revascularization (Supplementary Table S2). This finding may reflect event-driven prescribing, whereby patients maintained on long-term beta-blockers may represent a higher-risk subgroup with greater underlying cardiovascular risk. Important markers of disease severity such as detailed cardiac function, inflammatory burden, infarct burden, and completeness of revascularization were not available for adjustment, further amplifying residual bias. Thus, in our dataset, concurrent four-class medication use may have functioned primarily as a marker of higher underlying cardiovascular risk rather than an indicator of treatment effectiveness.

Furthermore, an additional methodological constraint is the ceiling effect specific to our tertiary-center sample: over 80% to 90% of participants were already receiving core preventive drugs (e.g., antiplatelets and statins), which restricts the statistical variance needed to identify incremental risk reduction through cross-sectional observation. Therefore, these findings should not be interpreted as evidence against the established efficacy of secondary prevention medications. Longitudinal registries collecting serial, granular cardiac functional and angiographic data are required to more accurately characterize individualized pharmacological benefits in future research.

### 4.3. Evolving Pharmacotherapy and Precision Medicine

The lack of association between the rigid “four-drug” definition of medication use and clinical outcomes may also reflect the increasing complexity of contemporary cardiovascular pharmacotherapy. Current clinical practice is gradually shifting away from a uniform “one-size-fits-all” approach toward more individualized treatment strategies.

For example, although dual antiplatelet therapy is routinely recommended after acute coronary syndromes, recent studies have explored de-escalation strategies for long-term management [24]. Similarly, the role of prolonged beta-blocker therapy in post-MI patients without heart failure or reduced ejection fraction has been increasingly debated in the recent literature [25,26]. In this context, some patients who discontinued certain medications over time may have been following clinically appropriate treatment adjustments rather than demonstrating poor adherence.

These considerations highlight an important methodological challenge for observational adherence studies. Measuring adherence solely by counting the number of medications may not fully capture the appropriateness or individualization of therapy in contemporary clinical practice. Future studies may benefit from incorporating more nuanced indicators of guideline-concordant and patient-specific pharmacotherapy.

### 4.4. Monitoring–Action Gap: Surveillance Versus Intervention

In our cohort, lipid monitoring was relatively frequent, with more than half of patients undergoing LDL-C testing at least every three months. However, monitoring frequency was not independently associated with lower odds of recurrent clinical events. This finding may reflect a potential gap between risk factor surveillance and therapeutic action.

Monitoring itself is primarily a diagnostic process and does not directly modify cardiovascular risk. Its clinical value depends on whether abnormal results prompt appropriate treatment adjustments, such as intensification of lipid-lowering therapy or reinforcement of lifestyle interventions.

Because our study did not capture detailed information on medication titration or treatment changes following laboratory testing, we were unable to evaluate whether monitoring results led to therapeutic interventions. Therefore, the lack of association observed in our analyses may reflect the complex relationship between surveillance, clinical decision-making, and treatment implementation in routine practice.

### 4.5. Interrelationships Among Domains of Secondary Prevention

We also examined a composite adherence score integrating medication use, health behaviors, and monitoring practices. Interestingly, this composite score was not significantly associated with clinical outcomes. This finding suggests that different domains of secondary prevention may contribute to cardiovascular risk through distinct pathways and may not operate in a simple additive manner.

Pharmacological therapies primarily target specific biological mechanisms involved in atherosclerotic disease, such as platelet aggregation and lipid metabolism [6]. In contrast, lifestyle behaviors influence a broader range of cardiometabolic factors, including body weight, metabolic health, and smoking exposure. These findings highlight the importance of considering multiple dimensions of secondary prevention simultaneously rather than relying on a single composite indicator of adherence.

### 4.6. Clinical and Public Health Implications

Young survivors of acute myocardial infarction represent a population with substantial long-term prevention needs that extend well beyond the initial hospitalization. The multidomain gaps observed in this study, particularly in healthy behaviors, suggest that routine secondary prevention for young AMI survivors should move beyond medication prescription alone and place greater emphasis on sustained behavioral support, structured follow-up, and long-term risk-factor management in everyday care. Given the observed association between better behavioral implementation and lower odds of recurrent myocardial infarction, strengthening lifestyle-oriented secondary prevention may be especially important in this population [27]. From a public health perspective, tailored implementation strategies that incorporate behavioral counseling, digital health tools, cardiac rehabilitation, and multidisciplinary collaboration may help address the psychosocial, occupational, and self-management challenges commonly faced by younger patients and support more sustained secondary prevention over time [28].

## 5. Limitations

This study has several limitations inherent to its design. First, the cross-sectional assessment of adherence and the retrospective ascertainment of clinical events preclude strict causal inference. Although we report statistical associations, the temporal relationship between adherence behaviors and clinical outcomes cannot be definitively established, and reverse causality cannot be excluded. It is highly plausible that experiencing a recurrent cardiovascular event served as a catalyst for subsequent positive behavioral changes (e.g., abrupt smoking cessation) or led to physician-directed intensification of pharmacological therapies. However, because the median interval from index AMI to the study visit was 6 years, the great majority of recurrent events occurred before the 12-month behavioral assessment window. A sensitivity analysis excluding the five participants whose events fell within the recall window confirmed the statistical robustness of the association, but it does not establish temporal precedence. These findings should therefore be interpreted as associations between recent behavioral implementation and a prior history of recurrent events.

Second, adherence to medications and lifestyle behaviors was assessed primarily through self-report, which may be subject to recall bias and social desirability bias and may therefore overestimate true adherence. In addition, self-reported data cannot capture important aspects of medication use such as dosage adequacy or intermittent non-adherence. Furthermore, our study did not collect data on critical psychosocial factors, such as psychological stress, depression, anxiety, and post-traumatic stress disorder. These psychological conditions are highly prevalent among young AMI survivors and can significantly impair medication adherence, smoking cessation, and physical rehabilitation [29]. The absence of these unmeasured psychosocial dimensions limits our ability to fully explore the underlying barriers to secondary prevention in this vulnerable population.

Third, pharmacological treatment was assessed as self-reported current medication use at the study visit. The lack of an independent inverse association between intensive medication use and clinical outcomes—and the observed trend toward higher risk with beta-blockers—must be interpreted with caution. This finding is heavily subjected to reverse causation (i.e., “event-driven prescribing,” where a recurrent event prompts physicians to intensify the drug regimen) and unmeasured residual confounding. To avoid model overfitting based on the limited number of outcome events, our multivariable models were restricted to approximately 12 covariates. Consequently, patients maintained on intensive four-class regimens likely differed in several important characteristics not captured in our models. Crucial unmeasured variables—such as Lipoprotein (a) levels, systemic inflammatory burden, precise left ventricular ejection fraction (LVEF), heart failure symptoms, and angiographic completeness of revascularization—could impart significant residual confounding by indication. Thus, current medication use in our study primarily reflects the intensity of treatment indication rather than true adherence, and our observational findings should under no circumstances be interpreted as a lack of efficacy for evidence-based secondary prevention drugs.

Fourth, selection bias and survivor bias are inherent to this observational design. By enrolling patients who survived at least 12 months after index AMI and attended outpatient follow-up at a single tertiary center, patients who suffered fatal recurrent events, those lost to follow-up, or those managed exclusively at primary care centers were excluded. Consequently, our findings are primarily representative of young AMI survivors who remain actively engaged in long-term care, which may limit generalizability to broader populations.

Fifth, the variable duration from index AMI to the study visit constitutes an important limitation. Participants with longer post-infarction follow-up had a longer window at risk for recurrent MI or repeat revascularization, which may inflate event prevalence in those with extended observation periods.

Sixth, our dietary adherence assessment used a simplified, non-validated 7-item instrument adapted from the AHA LE8 framework. While this approach was pragmatically necessary to ensure adequate statistical power in this high-risk cohort, it may not fully capture the complexity of dietary behavior, and the generalizability of our dietary findings warrants confirmation in future studies using validated dietary assessment tools.

Seventh, the overwhelming predominance of male participants (95.9%) reflects the true demographic distribution of premature coronary disease in China rather than sampling bias. Although a sensitivity analysis restricted to men confirmed the robustness of our findings, the small number of female participants precluded meaningful sex-stratified analyses or interaction testing. Consequently, our conclusions are primarily applicable to young male AMI survivors, and extrapolation to young women warrants caution.

Finally, there was a minor proportion of missing data regarding body mass index (8.5%), which necessitated a complete-case analysis for the fully adjusted models. However, given the low missing rate and the completeness of all other key variables, this is unlikely to have substantially biased our main conclusions.

## 6. Conclusions

In this hospital-based cross-sectional study of young survivors of acute myocardial infarction in China, substantial gaps were observed in the long-term implementation of multidomain secondary prevention, particularly regarding healthy behaviors. Better recent adherence to healthy behaviors was independently associated with lower odds of a prior history of recurrent myocardial infarction, whereas concurrent use of all four major medication classes and frequent lipid monitoring were not independently associated with recurrent outcomes. However, given the cross-sectional design and the fact that most recurrent events occurred before the behavioral assessment window, these findings represent associations between recent behavioral implementation and previous recurrent-event history. This study cannot establish temporal precedence or determine whether behavioral implementation reduced the subsequent risk of recurrent events. Nonetheless, these findings underscore the public health importance of strengthening lifestyle-oriented, multidisciplinary, and long-term follow-up strategies for young AMI survivors. Future longitudinal studies are needed to clarify these temporal relationships and to identify effective implementation strategies for sustained secondary prevention.

## Figures and Tables

**Table 1:** Characteristics of participants at the index AMI and at the study visit.

General Characteristics	Recurrent MI			Repeat Revascularization		
	Yes (*n* = 126)	No (*n* = 1251)	*p* Value	Yes (*n* = 188)	No (*n* = 1189)	*p* Value
Gender (Male)	125 (99.2)	1196 (95.6)	0.051	183 (97.3)	1138 (95.7)	0.293
Age (Y)	39.6 ± 3.9	39.1 ± 4.4	0.253	39.0 ± 4.2	39.1 ± 4.4	0.768
STEMI	107 (84.9)	962 (76.9)	0.039	154 (81.9)	915 (77.0)	0.129
Multi-vessel disease	65 (51.6)	526 (42.0)	0.039	109 (58.0)	482 (40.5)	<0.001
Hypertension	57 (45.2)	444 (35.5)	0.030	85 (45.2)	416 (35.0)	0.007
Diabetes	40 (31.7)	280 (22.4)	0.018	58 (30.9)	262 (22.0)	0.008
Overweight/obese	100 (79.4)	979 (78.3)	0.774	154 (81.9)	925 (77.8)	0.203
LDL-C >1.8 mmol/L	62 (49.2)	594 (47.5)	0.712	92 (48.9)	564 (47.4)	0.702
Healthy behavior						
Non-smoking/cessation	51 (40.5)	705 (56.4)	<0.001	91 (48.4)	665 (55.9)	0.054
Physical activity	86 (68.3)	944 (75.5)	0.076	132 (70.2)	898 (75.5)	0.119
Healthy diet	59 (46.8)	744 (59.5)	0.006	97 (51.6)	706 (59.4)	0.044
Adequate sleep	53 (42.1)	546 (43.6)	0.733	84 (44.7)	515 (43.3)	0.725
**Secondary preventive drugs**						
Antiplatelet drugs	116 (92.1)	1077 (86.1)	0.060	176 (93.6)	1017 (85.5)	0.002
Statins	111 (88.1)	987 (78.9)	0.014	166 (88.3)	932 (78.4)	0.002
Beta-blockers	88 (69.8)	736 (58.8)	0.016	134 (71.3)	690 (58.0)	<0.001
ACEI/ARB	53 (42.1)	439 (35.1)	0.120	73 (38.8)	419 (35.2)	0.340
**Interval of monitoring (M)**			0.995			0.743
≤1	11 (8.7)	107 (8.6)		15 (8.0)	103 (8.7)	
1–3	58 (46.0)	573 (45.8)		91 (48.2)	540 (45.4)	
>3	57 (45.2)	571 (45.6)		82 (43.6)	546 (45.9)	

**Table 2:** Utilization of guideline-recommended secondary prevention medications.

Medication Use	Initial Diagnosis of AMI	Outpatient Follow-Up	*p* Value
**Secondary preventive drugs**			
Antiplatelet drugs	1268 (92.1)	1193 (86.6)	<0.001
Statins	1200 (87.1)	1098 (79.7)	<0.001
Beta-blockers	1077 (78.2)	824 (59.8)	<0.001
ACEI/ARB	814 (59.1)	492 (35.7)	<0.001
**Number of drugs taken**			<0.001
0	89 (6.5)	137 (9.9)	
1	20 (1.5)	85 (6.2)	
2	153 (11.1)	300 (21.8)	
3	427 (31.0)	498 (36.2)	
4	688 (50.0)	357 (25.9)	

The antiplatelet regimen consisted of dual therapy upon the initial diagnosis of AMI, followed by monotherapy (aspirin, clopidogrel, or ticagrelor) during the outpatient follow-up phase; *p*-values were calculated using McNemar’s test for individual medication classes and the Wilcoxon signed-rank test for the number of drugs taken, comparing paired data from the initial AMI diagnosis and the outpatient follow-up.

**Table 3:** Secondary-prevention risk-factor monitoring patterns.

Item of Monitoring	Interval of Monitoring (M)			*p* Value
	≤1	1–3	>3	
LDL-C monitoring	118 (8.6)	631 (45.8)	628 (45.6)	0.055
LDL-C <1.8 mmol/L	54 (7.5)	351 (48.7)	316 (43.8)	
LDL-C ≥1.8 mmol/L	64 (9.8)	280 (42.7)	312 (47.6)	
BP monitoring	889 (64.6)	273 (19.8)	215 (15.6)	<0.001
Hypertension	409 (81.6)	63 (12.6)	29 (5.8)	
Without hypertension	480 (54.8)	210 (24.0)	186 (21.2)	
Blood glucose monitoring	425 (30.9)	457 (33.2)	495 (35.9)	<0.001
Diabetes	255 (79.7)	45 (14.4)	19 (5.9)	
Without diabetes	170 (16.1)	411 (38.9)	476 (45.0)	

**Table 4:** Characteristics associated with secondary-prevention implementation.

Characteristics	Medication Use			Good Behavioral Implementation			Frequent Lipid Monitoring		
	Yes (*n* = 357)	No (*n* = 1020)	*p* Value	Yes (*n* = 621)	No (*n* = 756)	*p* Value	Yes (*n* = 749)	No (*n* = 628)	*p* Value
Risk factors									
Gender (Male)	349 (97.8)	972 (95.3)	0.042	587 (94.5)	734 (97.1)	0.016	713 (95.2)	608 (96.8)	0.129
Age (Y)	38.7 ± 4.2	39.3 ± 4.4	0.047	39.9 ± 4.0	38.5 ± 4.6	<0.001	39.8 ± 4.1	38.3 ± 4.6	<0.001
STEMI	281 (78.7)	788 (77.3)	0.570	448 (72.1)	621 (82.1)	<0.001	589 (78.9)	480 (76.4)	0.328
Multi-vessel disease	168 (47.1)	423 (41.5)	0.066	284 (45.7)	307 (40.6)	0.056	337 (45.0)	254 (40.4)	0.089
Hypertension	191 (53.5)	310 (30.4)	0.001	218 (35.1)	283 (37.4)	0.371	266 (35.5)	235 (37.4)	0.464
Diabetes	92 (25.8)	228 (22.4)	0.188	133 (21.4)	187 (24.7)	0.147	202 (27.0)	118 (18.8)	<0.001
Overweight/obese	283 (79.3)	796 (78.0)	0.626	488 (78.6)	591 (78.2)	0.855	587 (78.4)	492 (78.3)	0.990
LDL-C level (mmol/L)									
LDL-C <1.8	196 (54.9)	525 (51.5)	0.264	327 (52.7)	394 (52.1)	0.842	405 (54.1)	316 (50.3)	0.165
Clinical outcomes									
Recurrent MI	43 (12.0)	83 (8.1)	0.028	38 (6.1)	88 (11.6)	<0.001	69 (9.2)	57 (9.1)	0.931
Revascularization	59 (16.5)	129 (12.6)	0.066	72 (11.6)	116 (15.3)	0.044	106 (14.2)	82 (13.1)	0.556

**Table 5:** Association between secondary-prevention implementation domains and clinical outcomes.

Clinical Outcome	Recurrent MI		Repeat Revascularization	
	OR (95%CI)	*p* value	OR (95%CI)	*p* value
Medication use				
Model 1	1.546 (1.047–2.283)	0.028	1.367 (0.978–1.911)	0.067
Model 2	1.522 (1.027–2.257)	0.036	1.305 (0.930–1.833)	0.123
Model 3	1.472 (0.979–2.212)	0.063	1.222 (0.861–1.736)	0.262
Good behavioral implementation				
Model 1	0.495 (0.333–0.735)	<0.001	0.724 (0.528–0.992)	0.044
Model 2	0.485 (0.324–0.726)	<0.001	0.717 (0.519–0.990)	0.043
Model 3	0.487 (0.324–0.731)	<0.001	0.723 (0.522–1.001)	0.050
Frequent lipid monitoring				
Model 1	1.016 (0.704–1.469)	0.931	1.089 (0.805–1.497)	0.556
Model 2	0.946 (0.648–1.378)	0.771	1.072 (0.779–1.474)	0.669
Model 3	0.928 (0.632–1.362)	0.702	1.059 (0.767–1.461)	0.729

Model 1: unadjusted. Model 2: adjusted for age, sex, AMI subtype, multivessel disease, and time since index MI. Model 3: further adjusted for hypertension, diabetes mellitus, overweight/obesity (defined as BMI ≥ 24 kg/m^2^), and LDL-C level, with mutual adjustment for the other adherence domains not under analysis. Models 1 and 2 included all 1377 participants; owing to missing BMI data in 117 participants (8.5%), Model 3 was performed using complete-case analysis (*n* = 1260). Abbreviations: OR, odds ratio; CI, confidence interval; MI, myocardial infarction; AMI, acute myocardial infarction; BMI, body mass index; LDL-C, low-density lipoprotein cholesterol.

## Data Availability

The data presented in this study are available on request from the corresponding author. The data are not publicly available due to ethical restrictions and the protection of research participants’ privacy.

## Supplementary Materials

The following supporting information can be downloaded at: https://www.mdpi.com/article/10.3390/healthcare14203407/s1, File S1: Supplementary Table: Table S1. Interrelationships among secondary-prevention domains. Table S2: Associations between individual secondary preventive medication classes and clinical outcomes (Fully Adjusted Model). Table S3. Multivariable logistic regression analysis of individual and cumulative health behaviors with recurrent cardiovascular events. Table S4. Sensitivity analysis for the association between secondary-prevention implementation domains and recurrent cardiovascular events after excluding participants with events during the 12-month recall window (*n* = 1372). Table S5. Sensitivity analysis restricting the study population strictly to male participants. File S2: Structured Questionnaire for Health Behaviors and Treatment Adherence in Young CHD Patients.

## Abbreviations

The following abbreviations are used in this manuscript:

AMI	Acute Myocardial Infarction
CAD	Coronary Artery Disease
PCI	Percutaneous Coronary Intervention
CABG	Coronary Artery Bypass Grafting
ACEI	Angiotensin-Converting Enzyme Inhibitor
ARB	Angiotensin Receptor Blocker
LDL-C	Low-Density Lipoprotein Cholesterol
Glu	Glucose; SBP: Systolic Blood Pressure
DBP	Diastolic Blood Pressure
OR	Odds Ratio
CI	Confidence Interval
SD	Standard Deviation
IQR	Interquartile Range
EMR	Electronic Medical Record
AHA	American Heart Association
CVD	Cardiovascular Disease
