# Supplementary material for: Long-Term Secondary Prevention Gaps Among Young Survivors of Acute Myocardial Infarction in China: A Hospital-Based Cross-Sectional Study
Source: Healthcare (Basel). 2026 Oct 13;14(20):3407. doi: 10.3390/healthcare14203407 (PMC13704252; doi:10.3390/healthcare14203407)
Supplement: Supplementary file 1 [file healthcare-14-03407-s001.zip › Supplementary File S1-Supplementary Table-format.pdf]

Supplementary Materials File S1: Supplementary Table

Supplementary Table S1. Interrelationships among secondary-prevention domains

Concurrent use of four medication classes	Behavioral implementation: No, n (%)	Behavioral implementation: Yes, n (%)	P value
No	572 (56.1)	448 (43.9)	0.138
Yes	184 (51.5)	173 (48.5)	
**Concurrent use of four medication classes**	**Frequent lipid monitoring: No, n (%)**	**Frequent lipid monitoring: Yes, n (%)**	**P value**
No	459 (45.0)	561 (55.0)	0.445
Yes	169 (47.3)	188 (52.7)	
**Behavioral implementation**	**Frequent lipid monitoring: No, n (%)**	**Frequent lipid monitoring: Yes, n (%)**	**P value**
No	360 (47.6)	396 (52.4)	0.098
Yes	268 (43.2)	353 (56.8)	

Supplementary Table S2: Associations between individual secondary preventive medication classes and clinical outcomes (Fully Adjusted Model).

Medication Class	Recurrent MI		Repeat revascularization	
	OR(95%CI)	P value	OR(95%CI)	P value
Antiplatelet drugs	1.068 (0.480–2.379)	0.872	1.508 (0.706–3.219)	0.289
Statins	1.428 (0.724–2.817)	0.304	1.152 (0.634–2.093)	0.643
Beta-blockers	1.434 (0.923–2.227)	0.109	1.652 (1.106–2.467)	0.014
ACEI/ARB	1.064 (0.706–1.604)	0.767	0.820 (0.560–1.202)	0.309

Note: Odds ratios (ORs) were derived from multivariable logistic regression models (Model 3) with simultaneous entry of all four medication classes. The models were additionally adjusted for age, sex, AMI subtype, multivessel disease, time since index MI, hypertension, diabetes mellitus, overweight/obesity (BMI $\geq$ 24 kg/m^2), LDL-C level, and mutual adjustment for behavioral and monitoring secondary-prevention implementation domains.

Supplementary Table S3. Multivariable logistic regression analysis of individual and cumulative health behaviors with recurrent cardiovascular events

Behavioral Metrics	Recurrent MI<br>Adjusted OR (95% CI)*	Revascularization<br>Adjusted OR (95% CI)*
Number of Ideal Behaviors (Ordinal)		
0–1 (Reference)	1.00	1.00
2 behaviors	0.739 (0.467–1.172)	0.748 (0.495–1.128)
3 behaviors	0.515 (0.313–0.849)	0.665 (0.436–0.953)
4 behaviors	0.166 (0.063–0.436)	0.438 (0.236–0.811)
Individual Behavioral Domains		
Smoking abstinence / Cessation	0.561 (0.382–0.825)	0.723 (0.526–0.995)
Physical activity	0.686 (0.451–1.044)	0.781 (0.545–1.119)
Healthy diet	0.622 (0.423–0.913)	0.710 (0.514–0.982)
Adequate sleep	1.008 (0.672–1.513)	1.207 (0.856–1.703)

* Fully adjusted Model 3: adjusted for age, sex, AMI type, multivessel disease, time from index AMI to enrollment, hypertension, diabetes, BMI, LDL-C, and mutually adjusted for other secondary-prevention implementation domains.

Supplementary Table S4. Sensitivity analysis for the association between secondary-prevention implementation domains and recurrent cardiovascular events after excluding participants with events during the 12-month recall window (n = 1,372).

Clinical outcome	Primary Analysis (n=1,377)		Sensitivity Analysis (n=1,372)*	
	OR(95%CI)	*P* value	OR(95%CI)	*P* value
**Recurrent MI**				
Medication use	1.472(0.979-2.212)	0.063	1.455(0.961-2.204)	0.078
Good behavioral implementation	0.487(0.324-0.731)	<0.001	0.517(0.343-0.799)	0.002
frequent lipid monitoring	0.928(0.632-1.362)	0.702	0.857(0.580-1.267)	0.430
**Repeat Revascularization**				
Medication use	1.222(0.861-1.736)	0.262	1.258(0.883-1.792)	0.203
Good behavioral implementation	0.723(0.522-1.001)	0.050	0.747(0.537-1.038)	0.083
frequent lipid monitoring	1.059(0.767-1.461)	0.729	1.003(0.724-1.390)	0.984

Note: Models were fully adjusted for age, sex, type of myocardial infarction, multivessel disease, time since index MI, hypertension, diabetes mellitus, overweight/obesity, LDL-C level, and mutual adjustment for other secondary-prevention implementation domains (Model 3). *Sensitivity analysis excluded 5 patients who experienced a recurrent clinical event within the 12-month recall window prior to the study visit.

Supplementary Table S5. Sensitivity analysis restricting the study population strictly to male participants

Clinical outcome	Overall population (n=1,377)		Male-only analysis (n=1,321)*	
	OR(95%CI)	P value	OR(95%CI)	P value
**Recurrent MI**				
Medication use	1.472(0.979-2.212)	0.063	1.494(0.993-2.249)	0.054
Good behavioral implementation	0.487(0.324-0.731)	<0.001	0.476(0.316-0.717)	<0.001
Frequent lipid monitoring	0.928(0.632-1.362)	0.702	0.920(0.625-1.354)	0.672
**Repeat Revascularization**				
Medication use	1.222(0.861-1.736)	0.262	1.267(0.889-1.805)	0.190
Good behavioral implementation	0.723(0.522-1.001)	0.050	0.693(0.497-0.965)	0.030
Frequent lipid monitoring	1.059(0.767-1.461)	0.729	1.076(0.776-1.492)	0.663

Note: Analysis restricted to male participants (n = 1,321). Models were fully adjusted for age, AMI subtype, multivessel disease, time since index MI, hypertension, diabetes mellitus, overweight/obesity (BMI ≥ 24 kg/m²), LDL-C level, and mutual adjustment for other secondary-prevention implementation domains.
