# Supplementary material for: Long-Term Secondary Prevention Gaps Among Young Survivors of Acute Myocardial Infarction in China: A Hospital-Based Cross-Sectional Study
Source: Healthcare (Basel). 2026 Oct 13;14(20):3407. doi: 10.3390/healthcare14203407 (PMC13704252; doi:10.3390/healthcare14203407)
Supplement: Supplementary file 1 [file healthcare-14-03407-s001.zip › Supplementary File S2-Structured Questionnaire for Health Behaviors and Treatment Adherence in Young CHD Patients-format.pdf]

Supplementary File S2: Structured Questionnaire for Health Behaviors and Treatment Adherence in Young CHD Patients

Questionnaire: Study on the Implementation of Health Behaviors and Secondary Prevention Among Young Patients with Coronary Heart Disease

Important interviewer instruction:
Unless otherwise specified, all questions regarding lifestyle habits, medication use, and monitoring frequency refer to the participant's typical, sustained status over the preceding 12 months prior to this visit. Interviewers must prompt participants to report their usual habits over the past 12 months, rather than transient conditions on the day of the interview.

Section 1. Basic Information

1 1 Name: ___________________________

2 Medical Record Number: ___________________________

3 Visit Date: ______ / ______ / ______

4 Sex: ☐ 1 = Male ☐ 2 = Female

5 Year of Birth: ______

6 Contact Phone Number: ___________________________

Section 2. Medical History

7. Diagnosis of Coronary Heart Disease (CHD):

☐ 1 = Angina pectoris☐ 2 = Myocardial infarction

7.1 Date of First Diagnosis: ______ Year ______ Month

7.2 Treatment Received: ☐ 1 = Medical therapy only☐ 2 = Percutaneous coronary intervention (PCI)

☐ 3 = Coronary artery bypass grafting (CABG)

8 History of Stroke: ☐ 1 = No☐ 2 = Ischemic stroke☐ 3 = Hemorrhagic stroke

9 History of Hypertension: ☐ 1 = No☐ 2 = Yes

9.1 Year of Diagnosis: ______

9.2 Use of Antihypertensive Medication: ☐ 1 = No☐ 2 = Yes

10History of Diabetes Mellitus: ☐ 1 = No☐ 2 = Yes

10.1 Year of Diagnosis: ______

10.2 Use of Antidiabetic Medication: ☐ 1 = No☐ 2 = Yes

Section 3. Lifestyle Habits

11.Height: ______ cm (1 decimal place)

12.Weight: ______ kg (1 decimal place)

13. Smoking Status:

☐ 1 = Never smoked

☐ 2 = Former smoker (cessation duration: ______ months)

☐ 3 = Current smoker

14.Alcohol Consumption:

☐ 1 = No

☐ 2 = Yes

14.1 Drinking Frequency: ______ times/week

14.2 Average Alcohol Intake per Occasion:

Baijiu (Chinese liquor): ______ mL

Red wine: ______ mL

Beer: ______ mL

Other: ______ mL

15.Salt Restriction (e.g., low-salt diet, reduced salt in cooking, avoiding pickled foods):

☐ 1 = Yes

☐ 2 = No

16.Oil Restriction (e.g., reduced animal fat, avoidance of fried foods, use of vegetable oils):

☐ 1 = Yes

☐ 2 = No

17.Frequency of Whole Grains and Mixed Beans Intake (>50 g per serving):

☐ 1 = 0 times/week

☐ 2 = 1–2 times/week

☐ 3 = 3–5 times/week

☐ 4 = >5 times/week

18.Average Daily Fruit Intake:

☐ 1 = >350 g

☐ 2 = 200–350 g

☐ 3 = <200 g

19.Average Daily Vegetable Intake:

☐ 1 = >500 g

☐ 2 = 300–500 g

☐ 3 = <300 g

20.Average Daily Red Meat Intake (pork, beef, lamb):

☐ 1 = >75 g

☐ 2 = 40–75 g

☐ 3 = ≤40 g or occasional

Interviewer note for dietary score calculation: *Seven specific dietary criteria are assessed based on the responses from Q15–Q20. One point is awarded for each satisfied criterion. A total score of ≥4 points is classified as achieving a "healthy diet" status.*

21.Weekly Egg Intake:

☐ 1 = 0

☐ 2 = 1–2

☐ 3 = 3–5

☐ 4 = >5

22.Frequency of Dairy Product Consumption:

☐ 1 = 0 times/week

☐ 2 = 1–2 times/week

☐ 3 = 3–5 times/week

☐ 4 = >5 times/week

23.Frequency of Eating Out (restaurants):

☐ 1 = 0 times/week

☐ 2 = 1–2 times/week

☐ 3 = 3–5 times/week

☐ 4 = >5 times/week

24.Daily Physical Activity Duration (walking, cycling, jogging, swimming, sports):

☐ 1 = <30 min

☐ 2 = 30–45 min

☐ 3 = 45–60 min

☐ 4 = >60 min

25.Average Daily Step Count:

☐ 1 = <6,000 steps

☐ 2 = 6,000–10,000 steps

☐ 3 = 10,000–15,000 steps

☐ 4 = >15,000 steps

Interviewer note for physical activity scoring: *The weekly moderate- to vigorous-intensity physical activity volume is calculated post-interview from the self-reported daily duration, frequency, and activity type. These are converted to MET values to determine if the participant meets the guideline*

threshold: ≥150 min/week of moderate-intensity OR ≥75 min/week of vigorous-intensity aerobic activity.

26.Average Sleep Duration per Night: ______ hours

27.Use of Sleep Medication:

☐ 1 = No

☐ 2 = Occasionally

☐ 3 = Frequently

Section 4. Current Secondary Prevention Treatment and Monitoring

28.Aspirin:

☐ 1 = No

☐ 2 = Yes

29.Other Antiplatelet Agents (e.g., clopidogrel, cilostazol, ticagrelor):

☐ 1 = No

☐ 2 = Yes

30.Statins:

☐ 1 = No

☐ 2 = Yes

31.ACE inhibitors (ACEI) / Angiotensin receptor blockers (ARB):

☐ 1 = No

☐ 2 = Yes

32.Beta-blockers:

☐ 1 = No

☐ 2 = Yes

33.Nitrates:

☐ 1 = No

☐ 2 = Yes

34.Calcium channel blockers:

☐ 1 = No

☐ 2 = Yes

35-1. Frequency of venous LDL-C (lipid) laboratory testing in the past 12 months:

☐ 1 = <1 month

☐ 2 = 1–3 months

☐ 3 = >3months

☐ 4 = Uncertain

35-2. Frequency of blood glucose testing (laboratory or home-measured) in the past 12 months

☐ 1 = <1 month

☐ 2 = 1–3 months

☐ 3 = >3months

☐ 4 = Uncertain

35-3. Frequency of blood pressure measurement (clinic- or home-measured) in the past 12 months:

☐ 1 = <1 month

☐ 2 = 1–3 months

☐ 3 = >3months

☐ 4 = Uncertain

36. Time Since Last Lipid/Glucose Blood Test: ______ months

Section 5. Effectiveness of Secondary Prevention

37.LDL-C level in the past 3 months: ______ mmol/L

38.Fasting Blood Glucose (Glu) in the past 3 months: ______ mmol/L

39.Blood Pressure in the past week:

SBP: ______ mmHg

DBP: ______ mmHg

40.Recurrent Myocardial Infarction:

☐ 1 = No

☐ 2 = Yes (Date: ______ Year ______ Month)

41.Repeat Revascularization:

☐ 1 = No

☐ 2 = Yes (Date: ______ Year ______ Month)

Section 6. Key Information at First Diagnosis of Acute Myocardial Infarction

42.Date of First CHD Diagnosis: ______ Year ______ Month

43.Type of Myocardial Infarction:

☐ 1 = STEMI

☐ 2 = NSTEMI

44.Extent of Coronary Lesions:

☐ 1 = Single-vessel disease

☐ 2 = Multi-vessel disease

45.LDL-C Level at Diagnosis: ______ mmol/L

46.Medications Prescribed at Diagnosis:

Aspirin: ☐ No ☐ Yes

Other antiplatelets: ☐ No ☐ Yes

Statins: ☐ No ☐ Yes

ACEI/ARB: ☐ No ☐ Yes

Beta-blockers: ☐ No ☐ Yes

Nitrates: ☐ No ☐ Yes

Calcium channel blockers: ☐ No ☐ Yes

Follow-up Information

Follow-up Date: ______ Year ______ Month
